# Prevalence of Preterm Birth in Saudi Arabia: A Systematic Review and Meta-Analysis

**DOI:** 10.7759/cureus.74562

**Published:** 2024-11-27

**Authors:** Eman H Almaghaslah, Israa Al Ibrahim, Sakinah S Al-Zahir, Ahmed Z Al Saif

**Affiliations:** 1 Preventive Health, Qatif Health Network, First Eastern Cluster, Ministry of Health, Qatif, SAU; 2 Preventive Medicine, The Joint Program of Preventive Medicine, Al-Ahsa, SAU; 3 Preventive Health, Rural Health Network, Central Division, Eastern Health Cluster, Ministry of Health, Safwa, SAU; 4 Keep Well, Model of Care, Eastern Health Cluster, Dammam, SAU

**Keywords:** maternal, pregnancy, premature birth, preterm, saudi arabia

## Abstract

Preterm birth, defined as delivery before 37 weeks of gestation, is a significant contributor to neonatal morbidity and mortality worldwide. Understanding the prevalence of preterm birth is critical to improving neonatal care, informing public health strategies, and supporting health care planning. The objective of this study was to explore the problem of preterm birth in Saudi Arabia by estimating the prevalence of preterm birth over a defined period of time. CINAHL, Cochrane Pregnancy and Childbirth Database, Embase, and Medline were searched, limiting the search to the human Saudi population, with no date or language restriction. Titles, abstracts, and full texts were screened to determine eligibility for inclusion. Included studies were assessed for risk of bias utilizing the Let Evidence Guide Every New Decision (LEGEND) tool. Then, data were extracted in a customized data collection form. Among the 14 full texts reviewed, 10 studies met the eligibility criteria and were included in the final review, with a total of 50,514 participants for singletons and 336 sets of twins or/and high-order gestation in different regions of Saudi Arabia. Six studies have been entered into the meta-analysis and resulted in a pooled prevalence of preterm birth of 7.89 per 100 live births (95% confidence interval: 6.94 to 8.97). For multiple pregnancies, the average prevalence of preterm birth was 91.3 per 100 live births (95% confidence interval: 88.3 to 94.3). The overall preterm birth rate in Saudi Arabia can be utilized in national health planning and public health policy development. By knowing the prevalence of preterm birth, healthcare practitioners and policymakers can effectively plan for capacity building and healthcare services to provide efficient and proactive care for preterm infants, ultimately improving patient outcomes by reducing neonatal morbidity and mortality.

## Introduction and background

Preterm birth, defined as delivery before 37 completed weeks of gestation, is the leading cause of neonatal mortality and the second leading cause of death in children under 5 years of age [1,2], the majority (60%) of which are late preterm births (34-37 weeks) [3-5]. Worldwide, an estimated 12-15 million preterm births occur annually, with approximately 1 million resulting in death from complications of prematurity [6].

Preterm birth is a multifactorial phenomenon that is now recognized to be the result of complex interactions among several factors. Most preterm births (75-85%) are either spontaneous or occur after premature rupture of membranes while the remaining 15-25% are iatrogenic [3,7,8]. Although the etiology of spontaneous preterm birth is multifactorial, infection is a common underlying cause. Additional risk factors, including sociodemographic, nutritional, biological, and environmental factors, have been shown to increase the risk of spontaneous preterm birth, but the precise mechanisms remain incompletely understood [6,7].

Preterm birth imposes a significant economic burden at both individual and societal levels. This impact is particularly pronounced in sub-Saharan Africa and Asia, which together account for approximately 60% of the world's preterm births [3]. In high-income countries, the financial impact is also substantial; for example, the United States alone allocated $25.2 billion to care for preterm infants in 2016 [9].

In Saudi Arabia, 54% of neonatal deaths were due to prematurity [10]. However, existing figures are projected or based on older data. This review aims to provide current evidence regarding the problem of preterm birth in Saudi Arabia, specifically to estimate the prevalence of preterm birth over a defined period of time as well as to inform public health strategies, to support health care planning, and to emphasize evidence-based practice in Saudi Arabia, as part of the Middle East and North Africa (MENA) region.

## Review

Methods

Protocol Registration and Reporting

The study protocol was registered at the International Prospective Register of Systematic Reviews (PROSPERO) under the registration number CRD42023405253. The report of this study follows the Preferred Reporting Items for Systematic Reviews and Meta-Analyses (PRISMA) guideline [11].

Eligibility Criteria

We included studies that reported preterm birth (defined as deliveries before completing 37 weeks of gestation) in Saudi Arabia. We did not specify any particular intervention or exposure to expand our search criteria, allowing for a broader inclusion of studies. The comparator group in our analysis was full-term births. Furthermore, we included all study designs except for reviews, non-human studies (genomic, molecular, chemistry, animals), case reports, and case series, as the latter two would not represent the general population. Additionally, we included peer-reviewed journal articles published up to June 30, 2023, with no language or date restrictions.

Information Sources

CINAHL, Cochrane Pregnancy and Childbirth Database, Embase, and Medline were searched. Gray literature, congress, and conference proceedings relating to preterm birth in Saudi Arabia were also searched.

Search Strategy

Our literature search was conducted using the following databases: CINAHL, Cochrane Pregnancy and Childbirth Database, Embase, and Medline from inception to June 30, 2023. Search terms included "Saudi," "preterm," "premature," "preterm birth," and relevant MeSH terms. Specific strategies combined these terms with Boolean logic connectors to target studies related to preterm birth in the Saudi Arabian population (see Appendices).

Study Selection and Data Collection Process

Studies were extracted from the databases and sent to screening software (www.Rayyan.ai) for independent screening of titles and abstracts by three reviewers: EA, IA, and SA. Deduplication was performed using the software's built-in deduplication function prior to screening. Then, the full-text screening was conducted in the same manner independently by the three reviewers. Any conflicts were resolved by discussion. Each included study was extracted into a Microsoft Excel spreadsheet (Microsoft Corporation, Redmond, WA, US), recording details such as author and year, city, study design, study duration, sample size, and study outcomes.

Data Items

All preterm births (spontaneous or induced) were not differentiated, as some articles did not specify the type and the reason for preterm delivery. All studies that defined preterm birth as childbirth before 37 weeks were included. Full-term birth, on the other hand, is any childbirth that occurs after 37 weeks of gestation. The location of the studies was verified via identified known hospital names in the regions and cities of Saudi Arabia.

Risk of Bias in Individual Studies

The included articles were assessed first for risk of bias/quality using the Let Evidence Guide Every New Decision (LEGEND) assessment tool [12], then graded for strength of evidence by the same tool. A summary figure of risk of bias was generated via the review manager software, Revman, version 5.1 (The Cochrane Collaboration, Copenhagen).

Analysis

The analysis of the pooled prevalence was conducted in SPSS version 29.0.2.0 (20) after calculating the standard error (SE) by using this formula for SE of proportion SE = sqrt(p*(1-p)/n), where (p) indicates the proportion of preterm prevalence and (n) indicates the total sample. The random-effect model was used, and inverse variance was utilized as the weight, including both within- and between-study variance. Moreover, the analysis used the restricted maximal likelihood to estimate the pooled prevalence of preterm birth as a preterm continuous outcome.

Summary Measures

The outcome measure of this study was the prevalence of preterm birth as a primary outcome. A summary was reported as a rate per 100 live births. The studies were summarized in the datasheet.

Results

Study Selection

**Figure 1:**
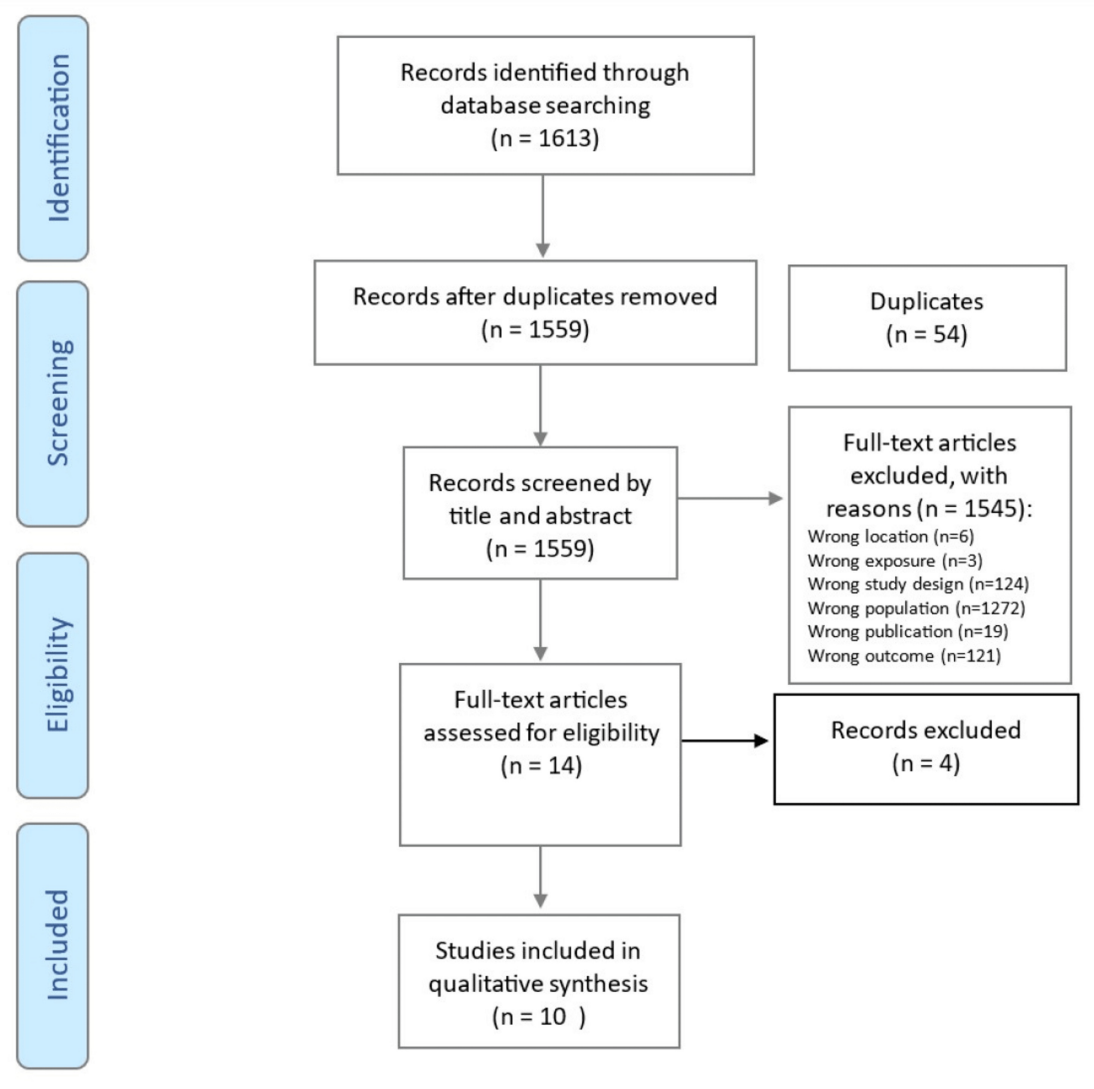
PRISMA flow chart PRISMA: Preferred Reporting Items for Systematic Reviews and Meta-Analyses

Database searches yielded 1613 studies. Following manual and electronic deduplication by EA and the three reviewers' title and abstract independent screening, 14 papers were selected for full-text screening for prevalence outcome. Studies were excluded from both screening rounds due to irrelevant location, exposure, study design, population, publication, or outcome criteria. The review yielded 10 publications suitable for quality assessment and analysis, displayed in the Preferred Reporting Items for Systematic Reviews and Meta-Analyses (PRISMA) flow diagram (Figure 1).

Study Characteristics

The final systematic review involved 10 papers, whereas the meta-analysis included 6. It included 1 cross-sectional study, 4 prospective cohorts, and 5 retrospective cohort studies, totaling 50,514 study participants for singletons and 336 sets of twins or multiple pregnancies in Saudi Arabia (Table 1).

**Table 1:** Study characteristics and grade of evidence m: month; y: year; 2a: good quality prospective cohort study; 3a: good quality retrospective cohort study

Author, Date	City	Method	Duration	Sample size (sets of multiple pregnancies)	Grade of evidence
Single pregnancy					
Serenius et al., 1988 [13]	Riyadh	Prospective Cohort	12 m (1979 - 1980)	1,149	2a
Al-Qurashi et al., 2016 [14]	Al-Khobar	Retrospective Cohort	5 y (June 2008 - June 2013)	6,455	3a
Wahabi et al., 2016 [15]	Riyadh	Multicenter Prospective Cohort	16 m (November 2013 - March 2015)	14,568	2a
Huseynova et al., 2021 [16]	Riyadh	Cross-Section	2 y, 4 m (1st March to 30th June 2017 - 2019)	7,226	3a
Fayed et al., 2022 [17]	Riyadh	Multicenter Prospective Cohort	16 m (November 2013 - March 2015)	13,403	2a
Asindi et al., 2002 [18]	Abha	Prospective Cohort	~ 11 y (March 1990 - Feb 2001)	7,713	2a
Twin pregnancy and high-order gestation					
Abu-Heija, 2003 [19]	Al-Khobar	Retrospective Cohort	10 y, 8 m (1st January 1990 - 31st August 2001)	(27) 88	3a
Al-Sunaidi and Al-Shahrani, 2011 [20]	Abha	Retrospective Cohort	3 y (January 2007 - December 2009)	(32) 96	3a
Mansouri and Ghazawi, 2007 [21]	Jeddah	Retrospective Cohort	20 Y (1^st^ January 1985 - 1^st^ February 2005)	(42) 56,206	3a
Algwiser et al., 1999 [22]	Riyadh	Retrospective Cohort	10 y (January 1987 - December 1996)	(236) 62,739	3a

The minimum study duration in Riyadh was 12 months, whereas the maximum was nearly 20 years in Jeddah. We did not include multiple pregnancy studies that reported a premature birth. The meta-analysis included only the first six studies to meet our objective of measuring the prevalence of preterm birth in singleton pregnancies, as adding the multiple pregnancy studies would not provide an accurate measure. Above all, the pooled prevalence was 7.89 per 100 live births, with a 95% confidence interval (CI) of 6.94-8.97 per 100 live births. There was a heterogeneity I-squared statistic of 95% and the Tau-square between the study variance test was 1.34.

Risk of Bias Within Studies

**Figure 2:**
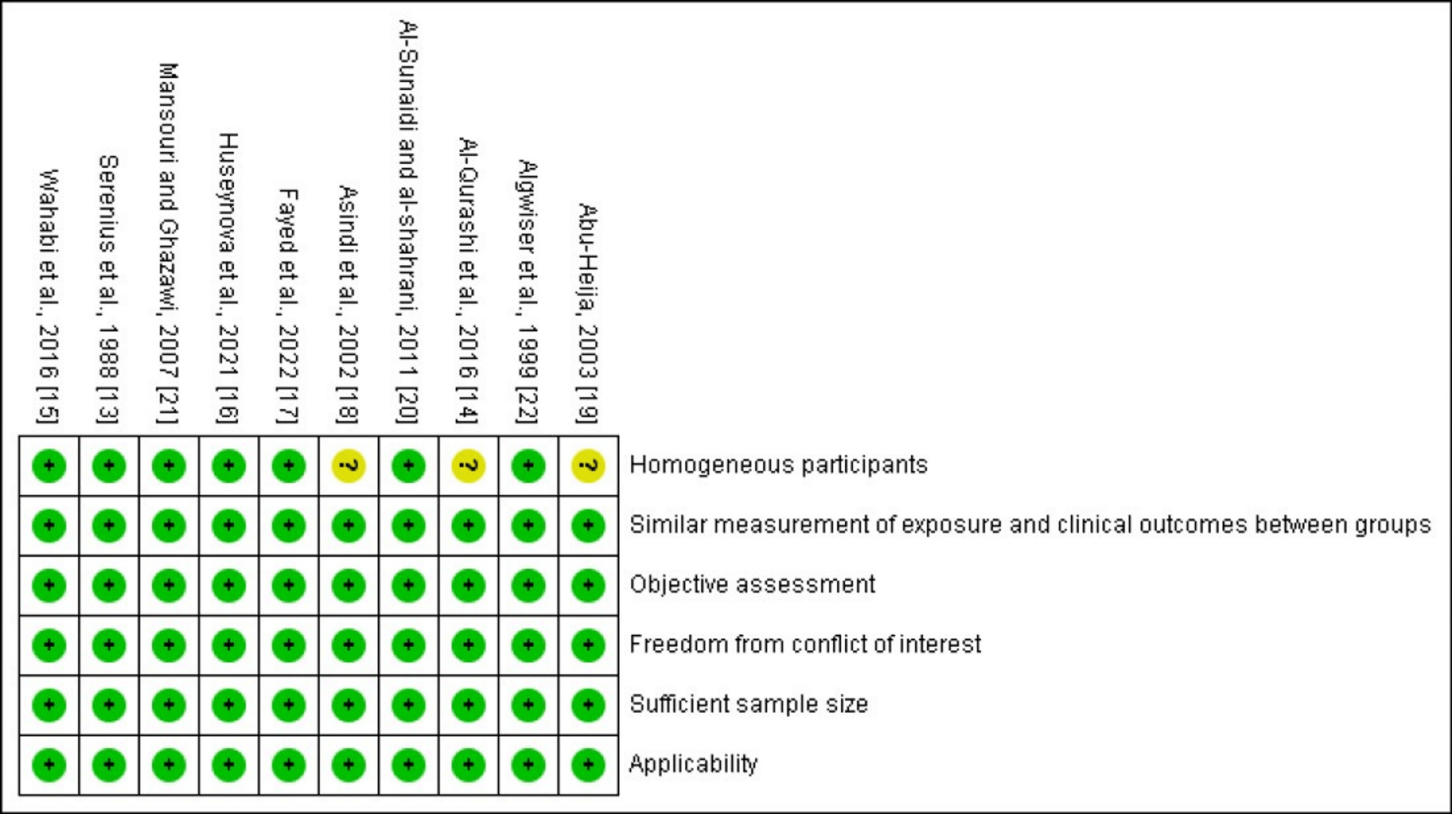
Risk of bias summary +: the criterion was fully met; ?: uncertainty or insufficient information to assess the criterion; yellow color: the study partially meets the criterion or has some concerns regarding the risk of bias; green color: the study fully meets the criterion with low risk of bias.

A low risk of bias resulted from the blinded reviewer's assessment of the studies, as illustrated in Figure 2.

Multiple Pregnancy and Preterm Birth

We had four studies that investigated preterm birth in multiple pregnancies. All were retrospective analyses of preterm birth medical records in major antenatal hospitals that could depict a better understanding of such a population. There were reporting differences in preterm definition. In one study, Algwiser et al. defined preterm birth as less than 34 weeks gestation [22]. The rest were less than 37-36 weeks [19-21]. The average prevalence of preterm birth in multiple pregnancies was around 91.3%, excluding the Algwiser et al. study (Table 2) [22].

**Table 2:** Summary of the preterm births among multiple pregnancies in Saudi Arabia

	Twins	Triplets	Quadruplets	Quintuplets	Total babies	Gestational age, week	Preterm Rate
Algwiser et al., 1999 [22]	598	28	6	-	1196	< 34	12.7%
Abu-Heija, 2003 [19]	-	21	3	3	88	< 37	96%
Mansouri and Ghazawi, 2007 [21]	-	36	5	1	133	< 37	88.1%
Al-Sunaidi and Al-Shahrani, 2011 [20]	-	32	-	-	96	< 36	90.6%

Synthesis of Results

**Figure 3:**
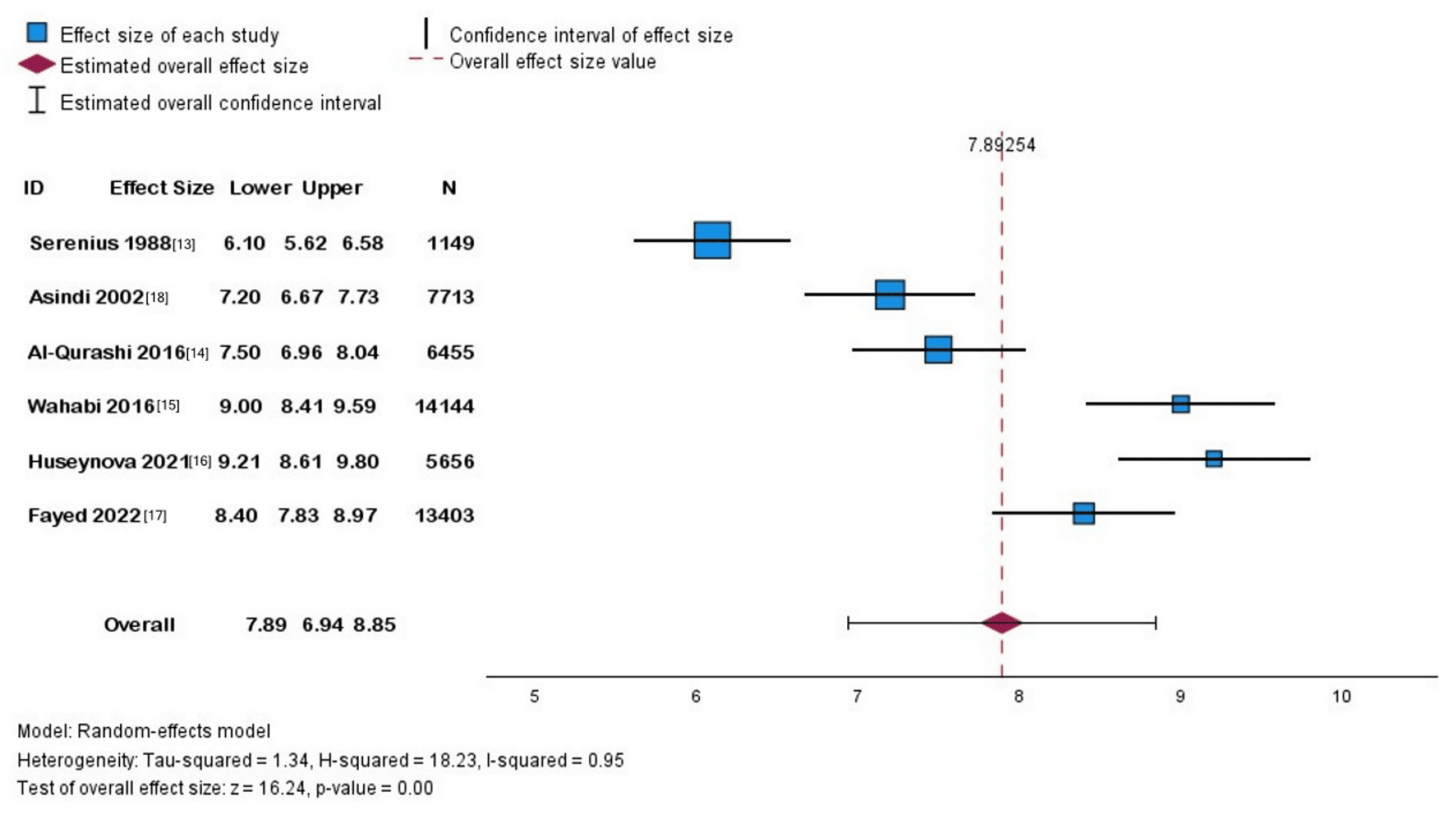
Forest plot for the prevalence of preterm births in Saudi Arabia

The included studies were summarized utilizing forest plots for singletons. A funnel plot was inappropriate for measuring publication bias since the included studies did not satisfy the required number, which is 10 studies, as per Cochrane recommendations (Figure 3) [23].

Additional Analysis

Meta-regression could not be performed due to the small number of studies that would be used to investigate the cause of high heterogeneity. However, we visually investigate whether there is a difference in the results based on the study duration or type of hospital, as the majority were from teaching/specialized centers in Riyadh [15,17,18,24] and Al-Khobar [14], except for the Abha study, which was conducted in a governmental public hospital [18]. There was no practical difference between them.

From the 14 papers with full-text assessed for eligibility, 10 were included in the current study and 4 were excluded for reasons shown in Table 3.

**Table 3:** Summary of studies excluded from this systematic review and meta-analysis

Number	Reference	Summary comment for exclusion
1	Serenius et al., 1988 [25]	Does not match our outcome, their outcome was neonatal death, and contains a mixed population
2	Srair et al., 1995 [26]	Does not match our outcome, and their outcome was neonatal death
3	Albahlol et al., 2020 [27]	Not representative population
4	Dallak et al., 2022 [28]	Self-reported outcome via face-to-face interview, and there was no data verification through hospital records

Discussion

In this study, we found an overall preterm birth prevalence of 7.89 per 100 live births and 91.3% for multiple pregnancies. A recent paper on the worldwide prevalence of all preterm births resulted in 9.9 per 100 live births in 2020 [29], aligning with our review.

These findings have important implications for national health planning and public health policy in Saudi Arabia. Knowing the prevalence of preterm birth allows health professionals and policymakers to plan for capacity building and provide efficient, proactive care for preterm infants. Recommended interventions include strengthening antenatal care programs, launching public awareness campaigns, implementing universal screening protocols, establishing specialized clinics for high-risk pregnancies, and investing in research to understand and prevent preterm birth, thereby improving maternal and neonatal health outcomes.

During the review, it was observed that the included studies were primarily hospital-based and relied on electronic medical records for data collection. While these studies provided valuable insights, there is a need for community-based surveys to accurately measure the prevalence of preterm birth in the general population. It is also important to note that the included studies were conducted in major cities, and the prevalence in rural areas may differ.

Moreover, having prevalence rates from specialized or teaching hospitals is reasonable as high-risk pregnancies are more likely to follow up in such healthcare facilities. However, having the prevalence from specialized centers will overestimate preterm birth more than the general population. Yet, the Asindi et al. study [18] was conducted in Abha Maternity Hospital, which had a preterm rate of 7.2, and that was consistent with the finding of Al-Qurashi et al. (7.5%) [14] and Serenius et al. (6.1%) [24] in teaching hospitals.

In our review, preterm birth was reported in Saudi Arabi first by Serenius et al. with the establishment of the neonatal intensive care unit in the King Faisal Specialist Hospital in Riyadh with an incremental trend of preterm birth over the years from 6.1% in 1980 till 9.2 by Huseynova et al. in 2017-2019 [16]. Adding the studies for one single pooled prevalence estimate might aid in healthcare planning; however, it would not provide a comprehensive picture of the changes over time.

Our analysis found that preterm birth rates among multiple pregnancies exceeded those reported in other countries' literature. We found sporadic measurement for preterm prevalence among twins; one study had a preterm birth rate of 62.1% at gestational age <37 weeks [30]. Furthermore, the high prevalence of preterm birth in multiple pregnancies in our result might be due to the population characteristics of the included studies. Three out of four included studies had a higher order gestation, meaning three and/or more infants in the study population, which has a higher risk of preterm birth than a twin pregnancy [19-21].

Strength and Limitations

To our knowledge, this is the first systematic review and meta-analysis investigating the prevalence of preterm births in Saudi Arabia. We faced a few limitations, such as that the published studies were only from major cities in the country, which could not represent the prevalence in rural areas. Furthermore, there was no consistency of preterm birth definitions in multiple pregnancies, which would have allowed us to perform a meta-analysis on such a population.

## Conclusions

Our study provides current evidence on the prevalence of preterm births in Saudi Arabia. By synthesizing data from different regions, we found that preterm birth remains a significant public health challenge. The findings highlight the importance of implementing standardized definitions and reporting practices in future research to ensure consistency and comparability. In addition, the high prevalence of preterm birth among multiple births calls for improved antenatal care and risk reduction intervention strategies. Policymakers and healthcare providers can use this information to inform public health strategies, improve healthcare planning, and ultimately improve outcomes for preterm infants in Saudi Arabia. Further research should continue to explore the factors that contribute to preterm birth and evaluate the effectiveness of interventions aimed at reducing these rates.

## Appendices

Search strategy

CINAHL

TX Saudi AND AB Preterm OR AB premature AND Subject: Major Heading AND

TX labor, premature

Cochrane, Embase

Saudi in All Text AND preterm in Title Abstract Keyword OR premature in Title Abstract Keyword - in Cochrane Reviews, Trials with 'Pregnancy and Childbirth', 'Neonatal' in Cochrane Groups (Word variations have been searched) Limited to Pregnancy & childbirth

Medline

("Preterm"[Title/Abstract] OR "premature"[Title/Abstract] OR "premature birth"[MeSH Terms]) AND ("saudi"[All Fields] OR "saudi arabia"[MeSH Terms])

## References

[1] Lawn JE, Gravett MG, Nunes TM, Rubens CE, Stanton C (2010). Global report on preterm birth and stillbirth (1 of 7): definitions, description of the burden and opportunities to improve data. BMC Pregnancy Childbirth.

[2] Dbstet A (1977). WHO: recommended definitions, terminology and format for statistical tables related to the perinatal period and use of a new certificate for cause of perinatal deaths. Acta Obstet Gynecol Scand.

[3] Goldenberg RL, Culhane JF, Iams JD, Romero R (2008). Epidemiology and causes of preterm birth. Lancet.

[4] Ahmed B, Abushama M, Konje JC (2023). Prevention of spontaneous preterm delivery - an update on where we are today. J Matern Fetal Neonatal Med.

[5] Beck S, Wojdyla D, Say L (2010). The worldwide incidence of preterm birth: a systematic review of maternal mortality and morbidity. Bull World Health Organ.

[6] Swarray-Deen A, Sepenu P, Mensah TE (2024). Preterm birth in low-middle income Countries. Best Pract Res Clin Obstet Gynaecol.

[7] Steer P (2005). The epidemiology of preterm labour. BJOG.

[8] Smid MC, Stringer EM, Stringer JS (2016). A worldwide epidemic: the problem and challenges of preterm birth in low- and middle-income countries. Am J Perinatol.

[9] Waitzman NJ, Jalali A, Grosse SD (2021). Preterm birth lifetime costs in the United States in 2016: an update. Semin Perinatol.

[10] (2012). Saudi Arabia, country profile. Maternal, newborn, & child survival. https://data.unicef.org/wp-content/uploads/country_profiles/SaudiArabia/Maternal_SAU.pdf.

[11] Liberati A, Altman DG, Tetzlaff J (2009). The PRISMA statement for reporting systematic reviews and meta-analyses of studies that evaluate health care interventions: explanation and elaboration. PLoS Med.

[12] (2012). LEGEND evidence evaluation tools & resources. James M Anderson Center for Health Systems Excellence. James M Anderson Center for Health Systems Excellence.

[13] Serenius F, Edressee AW, Swailem AR (1988). Characteristics of the obstetric population in a Saudi maternity hospital. Acta Paediatr Scand Suppl.

[14] Al-Qurashi FO, Yousef AA, Awary BH (2016). Epidemiological aspects of prematurity in the Eastern region of Saudi Arabia. Saudi Med J.

[15] Wahabi H, Fayed A, Esmaeil S (2016). Riyadh mother and baby multicenter cohort study: the cohort profile. PLoS One.

[16] Huseynova R, Bin Mahmoud L, Abdelrahim A (2021). Prevalence of preterm birth rate during COVID-19 lockdown in a tertiary care hospital, Riyadh. Cureus.

[17] Fayed A, Wahabi HA, Esmaeil S, Elmorshedy H, AlAniezy H (2022). Preterm, early term, and post-term infants from Riyadh mother and baby multicenter cohort study: the cohort profile. Front Public Health.

[18] Asindi AA, Archibong EI, Mannan NB (2002). Mother-infant colonization and neonatal sepsis in prelabor rupture of membranes. Saudi Med J.

[19] Abu-Heija AT (2003). Maternal and neonatal outcome of high order gestation. Arch Gynecol Obstet.

[20] Al-Sunaidi M, Al-Shahrani MS (2011). Fetomaternal and neonatal outcome of triplet pregnancy. Promising results. Saudi Med J.

[21] Mansouri HA, Ghazawi AH (2007). The maternal and neonatal outcome of high order gestation at King Abdulaziz University Hospital. Arch Gynecol Obstet.

[22] Algwiser A, Al Sultan S, Mesleh RA, Ayoub H (1999). Twin pregnancies: incidence and outcome - Riyadh Armed Forces Hospital experience. J Obstet Gynaecol.

[23] Collaborataion TC (2011). Recommendations on testing for funnel plot asymmetry. Cochrane Handbook for Systematic Reviews of Interventions.

[24] Serenius F, Edressee AW, Swailem AR (1988). Size at birth of infants in a Saudi maternity hospital. Acta Paediatr Scand Suppl.

[25] Serenius F, Swailem AR, Edressee AW, Ohlsson A (1988). Causes of perinatal death at a Saudi maternity hospital. Acta Paediatr Scand Suppl.

[26] Srair HA, Owa JA, Aman HA (1995). Cause-specific infant mortality rate in Qatif area, eastern province, Saudi Arabia. Ann Saudi Med.

[27] Albahlol IA, Almaeen AH, Alduraywish AA, Dar UF, El-Metwally TH (2020). Vitamin D status and pregnancy complications: serum 1,25-di-hydroxyl-vitamin D and its ratio to 25-hydroxy-vitamin D are superior biomarkers than 25-hydroxy-vitamin D. Int J Med Sci.

[28] Dallak FH, Gosadi IM, Haidar WN (2022). Prevalence of adverse birth outcomes and associated factors in Jazan, Saudi Arabia: a cross-sectional study. Medicine (Baltimore).

[29] Ohuma EO, Moller AB, Bradley E (2023). National, regional, and global estimates of preterm birth in 2020, with trends from 2010: a systematic analysis. Lancet.

[30] Li S, Gao J, Liu J (2021). Perinatal outcomes and risk factors for preterm birth in twin pregnancies in a Chinese population: a multi-center retrospective study. Front Med (Lausanne).

